# Nutritionally Optimized, Culturally Acceptable, Cost-Minimized Diets for Low Income Ghanaian Families Using Linear Programming

**DOI:** 10.3390/nu10040461

**Published:** 2018-04-07

**Authors:** Esa-Pekka A. Nykänen, Hanna E. Dunning, Richmond N. O. Aryeetey, Aileen Robertson, Alexandr Parlesak

**Affiliations:** 1 Institute for Nursing and Nutrition, Faculty of Health, Global Nutrition and Health, University College Copenhagen, Sigurdsgade 26, 2200 Copenhagen, Denmark; esapekka.nykanen@gmail.com (E.-P.A.N.); hannadunning@gmail.com (H.E.D.); aileen.robertson@foodconsult.info (A.R.); 2 Department of Population, Family and Reproductive Health, University of Ghana, LG 13 Legon, Accra, Ghana; raryeetey@ug.edu.gh

**Keywords:** linear programming, food baskets, non-communicable diseases, cost of diet, food accessibility

## Abstract

The Ghanaian population suffers from a double burden of malnutrition. Cost of food is considered a barrier to achieving a health-promoting diet. Food prices were collected in major cities and in rural areas in southern Ghana. Linear programming (LP) was used to calculate nutritionally optimized diets (food baskets (FBs)) for a low-income Ghanaian family of four that fulfilled energy and nutrient recommendations in both rural and urban settings. Calculations included implementing cultural acceptability for families living in extreme and moderate poverty (food budget under USD 1.9 and 3.1 per day respectively). Energy-appropriate FBs minimized for cost, following Food Balance Sheets (FBS), lacked key micronutrients such as iodine, vitamin B12 and iron for the mothers. Nutritionally adequate FBs were achieved in all settings when optimizing for a diet cheaper than USD 3.1. However, when delimiting cost to USD 1.9 in rural areas, wild foods had to be included in order to meet nutritional adequacy. Optimization suggested to reduce roots, tubers and fruits and to increase cereals, vegetables and oil-bearing crops compared with FBS. LP is a useful tool to design culturally acceptable diets at minimum cost for low-income Ghanaian families to help advise national authorities how to overcome the double burden of malnutrition.

## 1. Introduction

The Ghanaian population is experiencing a nutrition transition where a double burden of both undernutrition and an increasing prevalence of obesity and non-communicable diseases exist [1,2]. Malnutrition has remained the top cause of disability-adjusted-life-years in Ghana for the last twelve years [3]. 

Forty-two percent of women and 66% of children aged 6–59 months have either mild, moderate or severe anaemia [4]. The current level of iodine deficiency is considered to be “high and of major public health significance” by World Health Organization (WHO) standards [5]. Six percent of the women and 10% of the men are underweight (Body-mass index < 18.5) [4]. Nineteen percent of the Ghanaian children are stunted, with those residing in rural areas more likely to be stunted (22%) compared to those living in urban areas (15%) [4]. The consequences of stunting are high child mortality and morbidity, reduced cognitive development, productivity loss associated with care of sick children [6] and weakened national productivity [7]. Long term consequences include lower academic achievement, lower economic productivity in adulthood and poor maternal reproductive outcomes [8]. 

While undernutrition persists, simultaneously there is a growing prevalence of diet-related non-communicable diseases. Around one quarter of adults (27.8% of women and 21.8% of men) are overweight and 21.9% and 6.0% respectively are obese [9]. Increases in prevalence have been observed over the past 18 years and prevalence is higher in urban areas [9]. High prevalence of obesity leads to increased risk of hypertension, dyslipidaemia, type 2 diabetes and cerebrovascular diseases and is associated with a higher premature mortality [10]. For example, the incidence of both cerebrovascular and ischemic heart disease has increased by 15% each between 2005 and 2016 [3].

Intake of a diverse diet rich with micronutrients can help reduce the double burden of diet related diseases but lower income groups may not be able to afford the cost [11,12]. Around one third of the Ghanaian population live on USD 3.1 (Ghanaian Cedi, GHS 11.9) or less and one in eight on USD 1.9 (GHS 7.3) or less per day [13].

Linear programming (LP) is a useful tool for constructing fully nutritious and health-promoting diets optimized for cost-efficiency [14,15,16,17]. LP can be used to help advice policy makers which local foods can be recommended as being the most cost-effective [12]. The production of these foods can be supported by the government and their consumption promoted within the Dietary and Physical Activity Guidelines for Ghana [18,19]. Constraints based on national food consumption patterns can be built in to the analysis in an effort to achieve social acceptability [12,20].

The main goal of this study is to create a list of affordable foods (food baskets, FBs) that fulfil all Ghanaian nutrient recommendations while also helping to reduce risk of diet related non-communicable disease for low-income families of four. This can help authorities to design appropriate food production policies and refine their food based dietary guidelines.

## 2. Materials and Methods

### 2.1. Food Prices

Food prices were collected from a total of 11 urban market places and supermarkets in the Greater Accra, Central and Ashanti regions and from 8 rural market places from the Ashanti and Volta regions between February and April 2016. Depending on availability, prices were collected for a total of 266 food items in urban and 141 food items in rural areas. Between 1 and 11 prices for each food item were collected from supermarkets (urban only), corner stores, hawkers and market places. The median price of each item was used to minimize the effect of any outliers. Only the prices of raw or minimally processed foods were collected. Foods such as cheese, groundnut paste and local dishes such as “kenkey fante” (fermented ground corn), which were widely eaten, were included (Table S1). The prices were collected between February and April 2016 by local staff. Locals were hired to minimize any bias resulting by foods being more expensive if bought by non-Ghanaians. Information regarding the availability of wild foods, which could be harvested or collected at no financial cost, such as Jute (bush-okra) leaves, moringa leaves and giant African snails, was obtained from agriculture teachers working at the local Ngleshie Amanfro Senior High School.

### 2.2. Nutrient Contents

As much as possible, the West African food composition tables were used (Food and Agriculture Organization of the United Nations (FAO) [21]. Other sources used for nutrient composition included: Mozambican [22], American (SR28) [23], British (CoFID) [24], Danish (FoodData) [25], Finnish (Fineli) [26] and Norwegian [27] food composition databases. The nutrient values for cooked items were used when appropriate. The non-edible proportion of each food was calculated such as skin, stones and bones. Missing nutrient values were obtained from peer reviewed published articles [28,29] or by using values of similar varieties of foods. For processed foods (e.g., canned mackerel in tomato sauce) the nutrient values for each ingredient were calculated. 

### 2.3. Recommended Energy and Nutrient Intakes

The lower and upper boundaries for the recommended energy intakes (REIs) and recommended nutrient intakes (RNIs) for each family member were obtained from FAO and WHO (Table 1) [30,31]. The mean urban and rural household sizes in Ghana are 3.5 and 4.3, respectively [32]. Therefore, a Ghanaian food basket (FB) was created for an average family of four: 18–29.9-year-old woman (65 kg body weight and an assumed physical activity level of 1.75 (moderately active category [30])); 30–59.9-year-old man (65 kg bodyweight and an assumed physical activity level of 1.75); 5–5.9-year-old boy; and an 8–8.9-year-old girl. The estimates of body weight were based on the Ghanaian Demographic and Health Survey 2014 [4].

### 2.4. Optimization of the Local Food Baskets Using Linear Programming

Linear programming (LP) is an algorithm for maximizing or minimizing a given linear function for a set of constraints. LP is characterized by 3 features: the goal function, the decision variables and a list of linear constraints [33]. In the initial calculations, the goal function is the sum of the cost of each food in the FB; the decision variables consist of the weight of each food in the FB; and the linear constraints consist of the energy and nutrient recommendations (Table 1). To apply the algorithm to solve the LP system of inequalities, Microsoft Excel was used along with the open-source add-in OpenSolver [34].

The underlying objective function is defined as:

(1) $$GD=c_{0}+c_{1}X_{1}+c_{2}X_{2}+\;\cdots +c_{n}X_{n},$$

In Equation (1), *GD* stands for the goal determinant (e.g., total cost of the FB), *c* is the food-specific constant (e.g., specific cost in Cedi/kg food) and *X* is the amount of the corresponding food. Two different LP models with two different *GD*s were applied in the current study: either the total cost of the family member’s FB or the total departure from Ghanaian food supply patterns as reported by FAO’s Food Balance Sheets (see below) [35]. Both *GD*s, total cost and total deviation from prevailing food supply, were minimized. The implementation of the (non-linear) abs function into LP was done as described in detail by Darmon et al. [12].

All calculated models were subjected to constraints of nutritional adequacy, meaning that all nutrient contents of all reported food baskets were within the ranges as indicated in Table 1. The energy content of all FBs was set to meet the recommendations on energy intake by FAO/WHO [30].

Three different FBs were calculated, depending on urban or rural settings and whether or not wild foods were included:

1. An urban food basket (UFB): foods available in supermarkets and market places in the cities of Accra, Kasoa, Ngleshi Amanfro, Cape Coast and Kumasi.
2. A rural food basket (RFB): foods available in market places in the rural areas of Abono, Amedzofe, Vane, Fume and Kpedze.
3. A rural wild food basket (RWFB): foods available in rural market places, similar to RFB, plus locally available wild foods.

After the WHO/FAO energy and nutrient recommendations for each family member were satisfied (Table 1), the family’s FB was calculated by merging each of the 4 family members’ lists of foods into one FB. To optimize for social and cultural acceptability, the following two constraints were incorporated into the LP models that used the total cost as *GD* (Models i and ii):

- Groups of foods, in the FB, were constrained progressively into proportions that deviate as little as possible from those reported in the Food Balance Sheets (FBS) for Ghana from 2011 [35]. The total Relative Deviation (*RD*) for each food group was calculated as the sum of all relative differences between the FBS values minus the sum of foods contained in each food group in the FB:
  (2) $$RD=\sum_{i=1}^{n}\frac{\mathrm{abs}\left(m_{i}-M_{i}\right)}{M_{i}},$$
  In Equation (2), mi stands for the summed weights of all raw foods in a category, *M_i_* stands for the food supply of the corresponding category and *i* represents the running index of the categories (*n* = 31 in total, Table S1). The cost and the composition of the FB were calculated in a stepwise manner based on constrained maximum *RD*s per food group (200%, 100%, 70%, 50% and 40%). Constraining the *RD* resulted in a weight delimitation of the foods in the optimized FBs for each category (as indicated in Supplementary Table 1) that guaranteed a similarity to the prevailing FBS. The lower the delimiting value of *RD*, the more similar the resulting FB matched the food supply spectrum of the FBS [35].
- In order to prevent having to eat the same monotonous diet every day and to diversify the diet, the weight of a single food within its group was constrained to a maximum in a stepwise manner (200%, 100%, 70%, 50% and 40%). For example, to enforce a minimum of 3 different foods in a group, the contribution of each food was limited to below 50% of the food group’s weight.
- In a third model, the *GD* to be minimized for was set to be the total *RD*. When calculating the FBs that were most similar to the FAO’s Food Balance Sheets (FBS), the goal function of LP was changed to minimum absolute values of the sum of all *RD*s [12,15], while all nutritional constraints were maintained. Cost was constrained to rates of extreme and moderate poverty of USD 1.9 (GHS 7.3) or USD 3.1 (GHS 11.9) per adult per day [13] where about half of the average household income is spent on food and beverages [36]. To avoid unrealistically small amounts of food, which cannot be purchased, these FBs were calculated on a one-month basis.

## 3. Results

### 3.1. Cost-Minimized Food Baskets Similar to FBS to Fulfil Recommended Energy Intake (REI) Only

Cost-minimized FBs for Ghanaian families of four, similar to the Ghanaian FBS (maximum *RD* = 0%) and fulfilling all the REIs but without applying constraints for recommended micronutrient intakes, showed a relative shortfall of calcium, iodine, riboflavin, folate and niacin in both urban and rural settings within the ranges of 37–46%, 38–42%, 48–64%, 73–78% and 65–78%, respectively, depending on the setting. Vitamins A and B_12_ recommendations were met by 35% and 57% respectively but only in the urban basket. The recommended intake of iron for mothers was not achieved in any of these baskets (44–54% compared with recommendations).

### 3.2. Simple Optimized Food Basket Solutions

The nutrient shortfall described above could be overcome by using 12–13 foods (Table 2) at a total cost of GHS 6.4 and GHS 7.7 (USD 1.7 and USD 2.0) per day, respectively, for an urban (UFB) and rural (RFB) family of four. When wild local foods were incorporated into the RFB the cost could be reduced to GHS 4.2 (USD 1.1) per day (Table 2) with over two thirds of its total weight consisting of wild foods, mostly green leafy vegetables.

In both the UFB and RFB, beef liver was the only animal food selected and this contributed to less than 1% of their total weight. Sugar crops (i.e., sugar cane), oil-bearing and stimulating crops (i.e., coffee, fruits, products from live animals (e.g., eggs or milk), fish and seafood were not selected by LP for the simplest versions of the UFB and the RFB (Table 2). The RWFB contained considerable amounts of giant African snails (Table 2).

The micronutrients determining the cost of the FBs were the minimum recommendation levels for calcium, iodine, riboflavin, niacin, vitamins B12 and C along with the polyunsaturated fatty acids (Table 3). Similarly, the upper limit recommendations for protein, saturated fatty acids and total sugar constrained the cost. Vitamins A and E, folate and iron were constraints only in urban or rural settings (Table 3). Achieving the recommendations for these nutrients automatically meant that all the other nutrients listed in Table 1 were covered. Interestingly, if a higher recommended level of protein was permitted, the overall cost of the basket would drop.

### 3.3. Cost of Increasing Dietary Diversity

**Figure 1:**
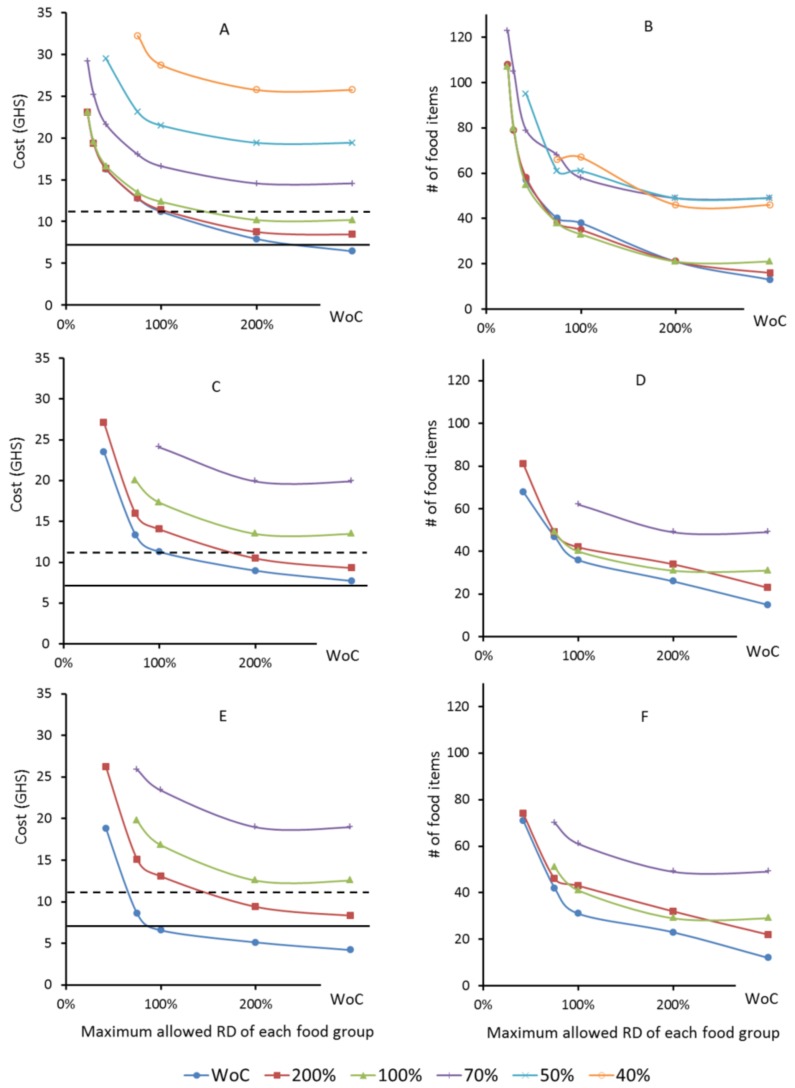
Cost (**A**,**C**,**E**) and diversity (**B**,**D**,**F**) of food baskets (FBs) for a Ghanaian family of four. Each point within the charts represents a nutritionally adequate FB. The FBs were optimized based on the foods available in the urban (UFB: urban food basket, Charts **A**,**B**) or in the rural environment (RFB: rural food basket, Charts **C**,**D**). Charts E+F build also on foods available in rural areas but include wild foods (RWFB: rural wild food basket). In the charts, each line represents a set of FBs that underlies the same constraint on the maximum contribution of a single food per food group (without constraint (WoC), 200%, 100%, 70%, 50% and 40%). Values on the X-axes indicate maximum allowed relative deviation (*RD*) from food group weights reported by most recent Ghanaian Food Balance Sheets [35]. The horizontal lines indicate threshold limits for poverty (USD 3.1, GHS 11.9, dashed) and extreme poverty (USD 1.9, GHS 7.3, solid). GHS, Ghanaian Cedi.

Only a few foods (11–14) are needed to cover all nutrient recommendations for one day (Table 2). Subsequently, the composition and cost of 69 additional FBs were calculated, all of which met the energy and nutrient recommendations. The proportion of each food was limited within each food group to ensure a greater daily variation. The number of foods in urban and rural FBs increased from 13 up to 108 and from 14 up to 68 respectively. If wild foods were incorporated into the rural basket the number of foods rose from 11 up to 71 (Figure 1). As the number of foods increased so did the cost: up to GHS 23.0, 23.5 and 18.8 (USD 6.0, 6.1 and 4.9) per day for the UFB, RFB and RWFB, respectively.

### 3.4. Alignment of the Food Baskets with Food Balance Sheets (FBS)

**Figure 2:**
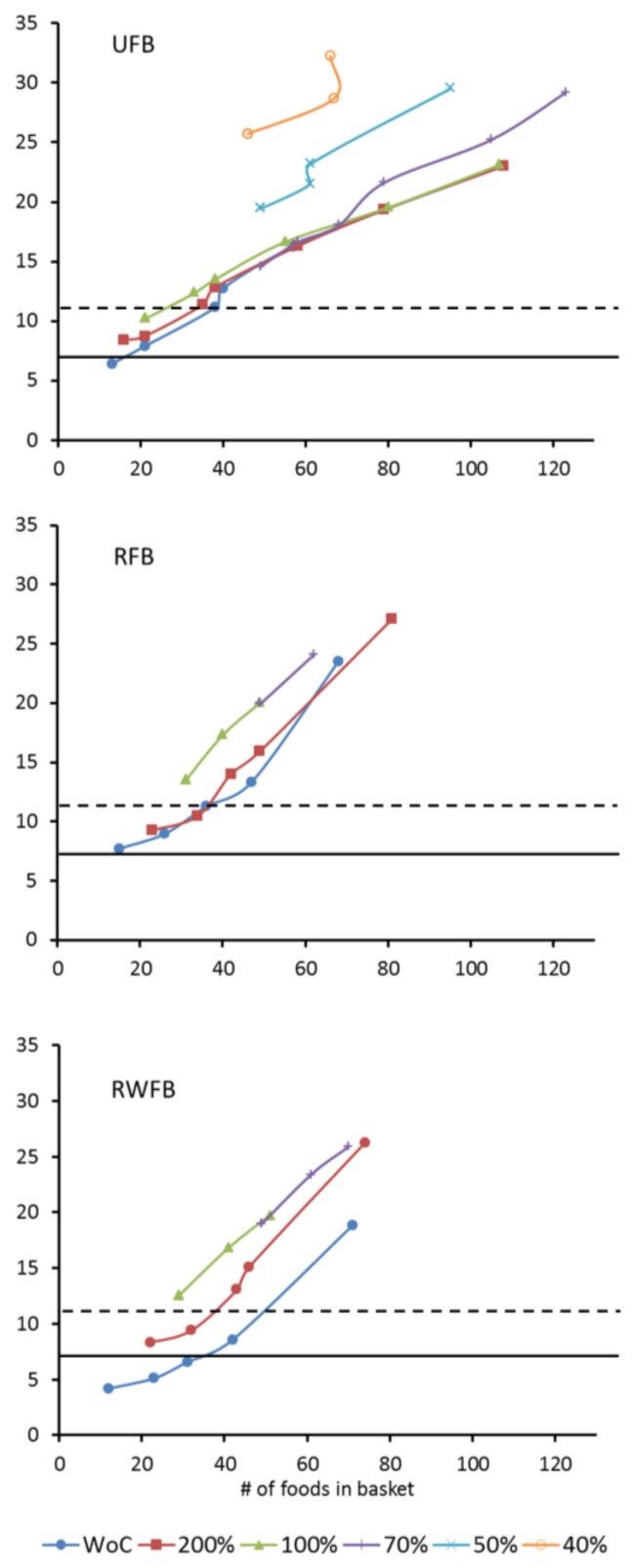
Correlation between cost and food diversity for single food baskets for a Ghanaian family of four. Charts refer to the UFB, the RFB and the RFB with wild foods included (RWFB). Each line represents a set of food baskets (FBs) that underlie the same maximum allowed contribution of a single food per category as described in the legend of Figure 1. The horizontal lines indicate threshold limits for poverty (USD 3.1, GHS 11.9, dashed) and extreme poverty (USD 1.9, GHS 7.3, solid).

Meeting all FAO and WHO nutrient recommendations for a family of four required a minimum of 23% averaged *RD* from the Ghanaian FBS in the urban areas, whereas in both of the rural scenarios a minimum of 42% averaged *RD* had to be allowed (Figure 1). Increasing the similarity of the FBs to the FBS was associated with a higher variety of foods and considerably increased cost (Figure 1). Thus, the more similar the FBs are to Ghanaian FBS, the more expensive they become (Figure 1) and a greater variety of food also results in higher cost (Figure 2).

The total energy per capita based on the Ghanaian FBS was 2940 kcal/day [35]. To achieve a feasible total weight of foods for each family member, the weight of each FBS category was standardized according to the corresponding recommended energy intake (e.g., 2550 kcal/day for the mother, Table 1). For example, according to the FBS the supply of maize and maize products per capita is 222 g per day, which standardized for the mother’s basket equalled 193 g per day (222 g × 2550 kcal/2940 kcal).

### 3.5. Food Baskets That Are Affordable for Families Living on Only USD 1.9 Per Day

FBs, calculated for budgets of GHS 7.3 (USD 1.9) per day per family and optimized for similarity with FBS, resulted in 22 foods being selected for the urban basket (Table 4). In rural areas, GHS 7.3 per day was not enough to cover the cost of a basket that would meet all energy and nutrient recommendations (Table 2) without including wild foods in the FBs. Inclusion of wild foods resulted in a FB consisting of 30 food items (Table 4). Cassava and maize products, such as banku flour, comprised about three quarters of the total weight of the UFB and animal foods contributed to less than 1% of the total weight. In rural areas, more than one quarter of the total weight came from wild foods such as mango, moringa and giant African snails. The share of animal foods in the RWFB was less than 4%.

### 3.6. Food Baskets That Are Affordable for Families Living on USD 3.1 Per Day 

Monthly FBs that were optimized for similarity with the FBS, on USD 3.1 (GHS 11.9) per day, comprised of 36, 31 and 39 foods for the UFB, RFB and RWFB respectively for a Ghanaian family of four (Table 5). The number of obtainable portions from the indicated weights can be calculated by dividing them by standard portion sizes for the corresponding foods as reported in the food composition databases [22,23,24,25,26,27].

**Figure 3:**
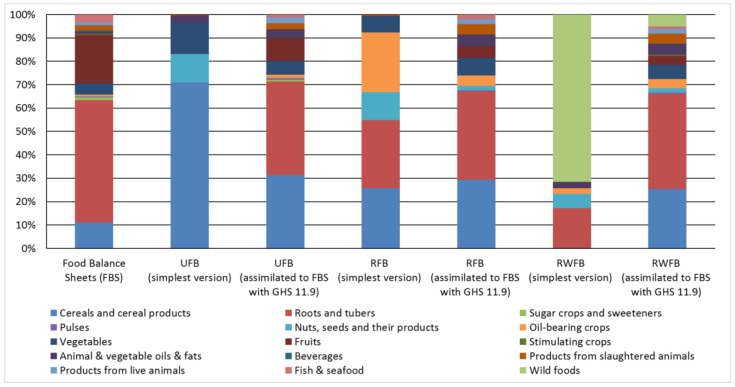
Food categories constituting the current food supply (left column), the simplest version of the UFB, RFB and RWFB and their more culturally acceptable forms that were aligned to current supply of food categories. The wild foods consisted of mango, dandelion, moringa, amaranth leaves, Jew’s mallow leaves, Giant African snails and waterleaves. FBS: Food Balance Sheets.

The food groups (cereals, roots and tubers) in these FBs were relatively more similar to the FBS compared with the less costly baskets (Figure 3). Cereals, roots and tubers contributed to around two thirds of total weight and consisted mainly of corn, cassava and their products whereas the amount of fruits was lower; and the amount of animal products and vegetable oils and fats was higher (Figure 3). 

## 4. Discussion

The nutrient analysis of the cost-minimized FBs that fulfilled only the recommended energy intakes but mirrored the Ghanaian Food Balance Sheets [35], highlights that the average Ghanaian food availability may be low in iron, iodine, vitamin A and folic acid (WHO/FAO recommendations). It is therefore not surprising to find a high prevalence of anaemia, goitre, night blindness, birth defects such as neural tube defects and stunting [4,32,37,38]. At the same time, the prevalence of noncommunicable diseases (NCDs) such as high blood pressure and cardiovascular disease is increasing in Ghana [39,40]. Considering the apparently low nutrient but high energy density of the Ghanaian food supply, it can be expected that the population will remain at risk of developing a double burden of malnutrition [1,2]. Government policies need to focus on how to leverage food-based solutions to eliminate micronutrient deficiencies and stunting in young children and reduce prevalence of obesity and NCDs in the population as a whole. 

Other authors have designed a linear programming diet model to determine the least costly basket of food items containing 13 food items that satisfy some of the recommended daily nutritional requirements of the average Ghanaian without addressing aspects of cultural acceptability and variety [41]. However one of the challenges of the LP methodology is that a limited number of foods are selected (Table 2) and this is likely to become too monotonous over a prolonged period and also not culturally acceptable [20]. In order to increase the chances of the FB being culturally acceptable it has been recommended to make the baskets as similar as possible to a population’s usual eating patterns [12,16] and to increase their dietary diversity [20]. Therefore, two constraints were enforced in our LP model to increase acceptability and diversity and both were associated with increasing cost (Figure 1 and Figure 2). A well-known barrier to being able to eat a healthy diet, is its cost [42]. This study aimed to identify what combination of foods can be purchased at a minimum cost while, at the same time, meeting the nutritional recommendations for a Ghanaian family of four and help to prevent diet-related diseases including obesity and NCDs.

The Ghanaian rural population appear to be more disadvantaged concerning the price of food compared with urban dwellers. For example, the least expensive nutritionally adequate and health promoting RFB for one day (Table 2) is 29% more expensive than the urban equivalent. FBs from urban settings, constrained to USD 3.1, were more diverse compared to their rural counterparts when no wild foods were included. When applying cost thresholds of USD 1.9 per person and day (extreme poverty) and assuming that 50% of the household’s income is spent on food, nutritionally adequate FBs could only be identified for the urban basket but not for the rural one. Adding only USD 0.11 (GHS 0.42) to the rural food budget would cover the cost of simplest FB for the rural population as well. Alternatively, under the USD 1.9 constraint, a nutritionally adequate solution in rural areas demands the inclusion of wild foods (Table 4). However, their use is limited due to: seasonality, local availability, considerable time consumption for harvest and their content of potentially toxic substances such as nitrates [43].

One potential to further reduce the cost would be to cultivate those foods that provide cost-delimiting nutrients, that is, iron, calcium, vitamin A, riboflavin, niacin and vitamin B_12_ and foods containing polyunsaturated fatty acids (Table 3). This could be for commercial purposes or subsistence farming or both. A further reduction in cost for nutritionally adequate FBs might be achieved by government subsidies such as the LEAP cash transfer program [44]. This would generate a possibility to further allow increased access to nutritious food by populations with limited financial resources. Such foods might be: dried soybeans and red whole-grain millet for iron; beef and chicken liver and dried false sesame leaves for vitamin A; iron and riboflavin, dried soya beans and dried false sesame leaves for folate [21].

Based on this study’s results, cost-effective, nutritionally adequate and healthy eating guidelines (Figure 3) for low-income Ghanaian families suggest reducing the intake of roots, tubers and fruits (with the exception of wild mango) and to increase the provision of cereals, vegetables and oil-bearing crops (e.g., soy and groundnuts), if available. For food categories where their availability might be relatively low in some areas (fish and seafood, sugar crops and sweeteners), the model suggests a further reduction. Of note, although all categories that were not cereals, roots and tubers contributed to only about third of the FB’s weight, they were essential to cover all RNIs.

The dietary patterns suggested by the models (Figure 3) match to a great extent the Dietary and Physical Activity Guidelines for Ghana [19]. Particularly the recommendations that a major part of a healthy diet should consist of cereals, roots and vegetables and a minor part should be fats, refined sugars and salts are mirrored in the results using LP. However, the relatively high intake of fresh fruit and moderate intake of meat, poultry, milk products and eggs recommended within Ghanaian FBDG appears to be too expensive for low-income families. The Ghanaian authorities may wish to review the dietary guidelines and consider what steps can be taken to alter the guidelines or help make healthy food more affordable to the population who are living under the poverty line. These steps can potentially avoid economic consequences of the current trajectory of the Ghanaian double burden of malnutrition [4].

The fact that all food prices were collected on-site guarantees local availability of the foods and a minimal feasibility of the suggested FBs. Some limitation of the validity of the results may arise from the fact that only the food supply (FBS) but not the food consumption was used as a reference point for cultural acceptability. Implementation of food consumption data of the Ghanaian population is desirable in further development of Ghanaian optimized FBs when those are available. Moreover, longitudinal studies on food availability and prices may contribute to the development of season-independent FBs with higher food variety. Intervention studies involving low-income groups would provide useful information concerning how practical the modelled food baskets are. Such studies should also take foods into consideration that have been cultivated in home gardens or received as a gift.

## 5. Conclusions

In Ghana, the prevalence of undernutrition (e.g., stunting and micronutrient deficiencies), obesity and diet related non-communicable diseases will increasingly affect vulnerable families with limited economic resources. The prevention of the double burden of malnutrition in Ghana will depend on healthy nutritious foods being culturally acceptable and readily available at affordable prices.

Using LP, culturally acceptable food baskets of minimum cost could be developed for urban and rural low-income Ghanaian families for a month when assuming that half of the household income is spent on food. These FBs fulfil all nutrient recommendations and, at the same time, can help to prevent obesity and diet-related NCDs. In addition, a further potential could be to reduce the prevalence of micronutrient deficiencies by promoting the production of more nutrient-dense foods that are rich in iron, folic acid, vitamin A, calcium, riboflavin, niacin and vitamin B_12_, plus foods rich in polyunsaturated fatty acids. Such foods might be soybeans, red millet, false sesame leaves as well as beef and chicken liver. The results were calculated using the local market prices so that findings could be helpful for the authorities to advise the entire food system including agriculture and horticulture sectors and the food producers and distributors, in order to support the local economy. 

## Figures and Tables

**Table 1:** Recommended energy intakes (REIs) and recommended nutrient intakes (RNIs) [29,30] applied as constraints for linear optimization of all calculated food baskets. SFAs, saturated fatty acids; n-3 and n-6 PUFAs, omega-3 and omega-6 polyunsaturated fatty acids; TFAs, trans-fatty acids; RAE, retinol activity equivalent. All recommendations refer to one day.

Age/Energy/Nutrient	Adult Female	Adult Male	Girl	Boy
Age (year)	18.0–29.9	30.0–59.9	8.0–8.9	5.0–5.9
Energy (kcal)	2550	2850	1698	1467
Protein (g)	54.0–95.6	58.0–106.9	26.2–63.7	17.1–55.0
Fat (g)	56.7–85.0	47.5–95.0	28.3–56.6	24.5–48.9
SFAs (g)	<28.3	<31.7	<18.9	<16.3
PUFAs (g)	17.0–28.3	19.0–31.7	11.3–18.9	9.8–16.3
n-3 PUFAs (g)	2.83–5.67	3.17–6.33	1.89–3.77	1.63–3.26
n-6 PUFAs (g)	14.17–22.67	15.83–25.33	9.43–15.09	8.15–13.04
TFAs (g)	<2.83	<3.17	<1.89	<1.63
Cholesterol (mg)	<300	<300	<300	<300
Carbohydrate available (g)	351–478	392–534	233–318	201–275
Fibre (g)	≥25.0	≥25.0	≥16.6	≥12.2
Total sugars (g)	<31.9	<35.6	<21.2	<18.3
Na (mg)	<2000	<2000	<2000	<2000
K (mg)	≥3510	≥3510	≥2337	≥1688
Ca (mg)	≥1000	≥1000	≥700	≥600
Mg (mg)	≥220	≥260	≥100	≥76
Fe (mg)	≥29.4	≥13.7	≥8.9	≥6.3
Zn (mg)	≥4.9	≥7.0	≥5.6	≥4.8
Se (µg)	≥26	≥34	≥21	≥22
Iodine (µg)	≥150	≥150	≥120	≥90
Vit A-RAE (µg)	≥500	≥600	≥500	≥450
Thiamine (mg)	≥1.10	≥1.20	≥0.90	≥0.60
Riboflavin (mg)	≥1.10	≥1.30	≥0.90	≥0.60
Vit B6 (mg)	≥1.30	≥1.30	≥1.00	≥0.60
Vit B12 (µg)	≥2.40	≥2.40	≥1.80	≥1.2
Vit C (mg)	≥45.0	≥45.0	≥35.0	≥30.0
Vit E (mg)	≥7.5	≥10.0	≥7.0	≥5
Folate (µg)	≥400	≥400	≥300	≥200
Niacin (mg)	≥16.0	≥14.0	≥12.0	≥8.0

**Table 2:** Simplest versions of Ghanaian food baskets for the urban environment (UFB, 12 foods), for the rural environment (RFB, 13 foods) and for the rural environment including wild foods (RWFB, 12 foods). During the cost optimization by linear programming, these food baskets were not constrained to achieve any forms of diversity or similarity to current food supply patterns. GHS, Ghanaian Cedi.

Category	Food Item	UFB		RFB		RWFB	
		Weight (g)	Cost (GHS)	Weight (g)	Cost (GHS)	Weight (g)	Cost (GHS)
Cereals and cereal products	Banku flour	551	1				
	Maize, white, flour refined			61	0.2		
	Millet, red, whole grain	1255	2.55	546	1.39		
	Oats, dried	36	0.28	31	0.37		
	Wheat, whole grains	63	0.25				
Roots and tubers	Cassava, flour, gari, yellow			726	1.63	900	2.03
Legumes	Cowpea, white, dried	2	0.01				
	Groundnut, dried			68	0.54	21	0.17
	Groundnut, paste			1	0.01		
	Groundnut, roasted					35	0.32
	Soy bean, flour			568	1.9	69	0.23
Nuts, seeds and their products	Coconut, whole, immature kernel, fresh	333	0.21	297	0.19	318	0.2
Vegetables and their products	Okra, dried, grounded			112	1.08		
	Cabbage, green	229	0.5				
	Cocoyam leaves			59	0.11		
	False sesame (yaudo) leaves, dried	129	0.72				
Animal & vegetable oils & fats	Soy oil	26	0.29				
	Vegetable oil	58	0.37	6	0.04	144	1.13
Products from slaughtered animals	Beef liver	10	0.21	14	0.2	4	0.06
Miscellaneous	Salt, iodized	17	0.04	17	0.06	15	0.05
Wild foods	Dandelion					744	0
	Jew’s mallow (ademe) leaves					1726	0
	Giant African snails					938	0
	Water leaves					329	0
	Sums	2709	6.44	2507	7.73	5243	4.19

**Table 3:** Nutrients that work as active constraints on the cost of Ghanaian food baskets. A shaded cell indicates an active constraint for the UFB (U; lower limit, orange; upper limit, blue), the RFB (R; lower limit, beige; upper limit, green) or the RWFB (W; lower limit, brown; upper limit, violet). Lowering the lower limits and increasing the upper limits of active constraints would result in reduced cost. SFA, saturated fatty acids; PUFA, polyunsaturated fatty acids; Vit, vitamin.

Limits	Family Member	Macronutrients															Minerals						Trace Elements						Vitamins																				
		Protein			SFA			n-3 PUFA			n-6 PUFA			Total Sugar			Sodium			Calcium			Iron			Iodine			Vit A			Riboflavin			Niacin			Folate			Vit B_12_			Vit C			Vit E		
		U	R	W	U	R	W	U	R	W	U	R	W	U	R	W	U	R	W	U	R	W	U	R	W	U	R	W	U	R	W	U	R	W	U	R	W	U	R	W	U	R	W	U	R	W	U	R	W
Lower limits	Mother																																																
	Father																																																
	Girl																																																
	Boy																																																
Upper limits	Mother																																																
	Father																																																
	Girl																																																
	Boy																																																

**Table 4:** Composition of a monthly urban food basket (UFB, 22 foods) and rural food basket including wild foods (RWFB, 30 foods) costing GHS 7.3 (USD 1.9) per day which has been optimized for similarity towards the most recent food supply patterns from FAOs food balance sheets [35]. GHS 7.3 per day did not cover the cost for a rural food basket without including wild foods.

Food Category	Food	UFB		RFB		RWFB	
		Mass (kg)	Cost (GHS)	Mass (kg)	Cost (GHS)	Mass (kg)	Cost (GHS)
Cereals & cereal products	Banku flour	46.33	84.2	No solutions below 7.31 GHS/day			
	Bread, “Sugarbread”					0.19	1.0
	Maize, white, flour refined					5.06	16.7
	Maize, yellow, whole kernel, dried	0.59	1.4			1.36	5.5
	Millet, red, whole grain	3.40	6.9			2.45	6.2
	Oats, dried	1.28	10.2			0.33	4.0
	Pearl millet, whole grain (with bran)					1.09	2.9
	Wheat flour, white					1.82	7.7
	Wheat, whole grain	5.36	21.1				
Roots and tubers	Cassava, flour, gari, yellow					38.72	87.2
	Cassava, tuber, raw	16.26	25.6				
Sugar crops and sweeteners	Sugar					0.17	0.6
Pulses	Cowpea, white, dried	0.64	2.3				
	Cowpea, red, dried					0.46	2.0
Nuts, seeds and their products	Coconut, immature kernel fresh kernel, fresh	1.00	0.6			1.46	0.9
	Colanut					0.24	1.9
Oil-bearing crops	Groundnut, dried					2.58	20.3
	Soya bean, dried	0.09	0.4				
	Soya bean, flour	0.26	1.9			4.27	14.3
Vegetables and their products	False sesame, leaves, dried	3.23	18.1				
	Onion, red	0.36	1.0				
	Onion, white					1.75	5.0
Fruits	Banana, white flesh	0.45	1.1			0.46	0.6
	Lemon					0.41	2.7
	Papaya, ripe	2.25	5.4				
Stimulating crops	Coffee, instant, powder	0.01	1.9			0.02	4.4
Animal & vegetable oils & fats	Soya oil	1.26	14.2				
	Vegetable oil	1.86	11.9			2.71	21.3
Products from slaughtered animals	Beef liver	0.34	6.7			0.14	2.0
	Beef, kidney					0.02	0.2
	Beef, meat, lean, boneless					0.30	4.3
	Beef, thigh, boneless	0.29	2.6				
	Chicken, liver					0.22	2.4
Products from live animals	Butter, from cow’s milk (without salt)	0.03	0.9				
	Egg, chicken	0.29	2.5			0.30	2.9
Fish & seafood	Whitefish, frozen					0.52	3.4
Miscellaneous	Salt, iodized	0.54	1.4			0.52	1.9
Wild foods	Giant African snails, with shell					1.94	0.0
	Mango					3.57	0.0
	Moringa leaf					17.46	0.0
	Water leaf					0.25	0.0
	Total	86.12	222.41			90.78	222.41

**Table 5:** Composition of a monthly urban food basket (UFB, 36 foods), rural food basket without wild foods (RFB, 31 foods) and rural food basket including wild foods (RWFB, 39 foods) costing GHS 11.9 (USD 3.1) per day which has been optimized for similarity towards the most recent food supply patterns from FAOs food balance sheets [35].

Food Category	Food Item	UFB		RFB		RWFB	
		Mass (kg)	Cost (GHS)	Mass (kg)	Cost (GHS)	Mass (kg)	Cost (GHS)
Cereals & cereal products	Banku flour	11.29	20.5				
	Bread, “Sugarbread”	1.48	7.8	3.65	20.1	2.78	15.4
	Bread, wheat, white	0.89	3.5				
	Kenkey fante, maize version					0.57	0.9
	Maize, white, flour refined			6.42	21.2	4.53	15.0
	Maize, yellow, whole kernel, dried	4.19	9.7			1.32	5.4
	Millet, red, whole grain	1.36	2.8	4.06	10.3	1.92	4.9
	Millet, whole grain, flour (with bran)	1.88	11.7				
	Oats, dried, raw	0.03	0.2	0.48	5.8	0.48	5.8
	Pearl millet, whole grain (with bran)			0.94	2.5	0.94	2.5
	Rice, brown			4.18	22.1	4.18	22.1
	Rice flour, white	2.47	13.1				
	Rice, Thai fragrant	5.93	26.2				
	Rice, white			4.42	16.6	4.42	16.6
	Wheat flour, white			1.03	4.3	1.89	8.0
	Wheat, whole grain	2.80	11.0				
Roots and tubers	Cassava, tuber	27.41	43.2				
	Cassava, flour, gari, white	13.87	52.3				
	Cassava, flour, gari, yellow			23.55	53.0	25.46	57.3
	Cocoyam, tuber			8.91	16.8	8.91	16.8
	Water yam, tuber					2.41	3.1
Sugar crops & sweeteners	Sugar, white	0.62	2.6				
Legumes and their products	Cowpea, white, dried	0.64	2.3				
	“Okogrono,” bean, dried			0.46	2.9	0.46	2.9
Nuts, seeds and their products	Cashew nut	0.23	12.4				
	Coconut, immature kernel			1.46	0.9	1.46	0.9
	Colanut			0.24	1.9	0.24	1.9
Oil-bearing crops	Groundnut, dried	0.95	6.9				
	Soya bean, dried	0.40	2.1				
	Soya bean, flour			3.84	12.8	3.64	12.2
Vegetables	False sesame, leaves, dried	4.54	25.5				
	Okra, dried, grounded			4.56	43.8	3.84	36.9
	Onion, red	1.71	4.7				
	Onion, shallot			0.14	0.6		
	Onion, white			1.61	4.6	1.75	5.0
Fruits	Banana, white flesh	0.45	1.1	0.46	0.6	0.46	0.6
	Grapefruit	5.70	14.2				
	Lemon			0.41	2.7	0.41	2.7
	Papaya, ripe			3.57	2.4	2.52	1.7
	Watermelon	3.90	6.7				
Stimulating crops	Coffee, instant, powder	0.02	2.4	0.02	4.4	0.02	4.4
	Cocoa, powder					0.37	13.7
Animal & vegetable oils & fats	Vegetable oil	2.49	15.9	4.18	32.9	4.38	34.5
	Soya oil	0.93	10.4				
	Palm oil, refined	0.39	2.0				
Products from slaughtered animals	Beef liver	0.32	6.4				
	Beef, meat, lean, boneless			0.30	4.3	0.30	4.3
	Beef, thigh, boneless	0.29	2.6				
	Beef, tripe, frozen	0.07	0.7				
	Chicken, back, with bone, frozen			2.01	12.7	2.01	12.7
	Chicken, leg, with bone, frozen	1.97	12.6				
	Chicken, liver					0.11	1.2
	Goat, liver			1.00	22.1	0.89	19.6
	Pig, foot, frozen			0.57	7.2	0.48	6.1
Products from live animals	Butter, (cow’s milk), unsalted	0.03	0.9				
	Egg, chicken	0.29	2.5	0.30	2.9	0.30	2.9
	Milk, UHT, banana flavour flavoured, 3.2% fat	2.32	17.4	1.50	16.6	1.50	16.6
Fish & seafood	African ghost crab, whole, fresh	0.83	4.6				
	Tuna, whole, fresh	0.36	2.5				
	Mudfish, dried, salted			1.59	11.3	0.43	3.0
	Whitefish, frozen			0.12	0.8	0.54	3.6
Miscellaneous	Salt, iodized	0.50	1.3	0.48	1.7	0.50	1.8
Wild foods	Amaranth leaves					2.62	0.0
	Giant African snails, with shell					0.57	0.0
	Mango					1.05	0.0
	Moringa leaves, fresh					0.43	0.0
	Total	103.54	362.8	86.46	362.8	91.11	362.8

## Supplementary Materials

The following are available online at http://www.mdpi.com/2072-6643/10/4/461/s1. Table S1: List of included Ghanaian foods.

## References

[1] Doku D.T., Neupane S. (2015). Double burden of malnutrition: Increasing overweight and obesity and stall underweight trends among Ghanaian women. BMC Public Health.

[2] Tuoyire D.A., Kumi-Kyereme A., Doku D.T. (2016). Socio-demographic trends in overweight and obesity among parous and nulliparous women in Ghana. BMC Obes..

[3] Institute for Health Metrics and Evaluation Ghana. http://www.healthdata.org/ghana.

[4] Ghana Statistical Service, Ghana Health Service, The DHS Program ICF International (2015). Ghana Demographic and Health Survey 2014. Key Indicators. http://www.statsghana.gov.gh/docfiles/DHS_Report/Ghana_DHS_2014-KIR-21_May_2015.pdf.

[5] Ghana Statistical Service, Ghana Health Service, The DHS Program ICF International (2015). Ghana Demographic and Health Survey 2014. https://dhsprogram.com/pubs/pdf/FR307/FR307.pdf.

[6] World Health Organization (WHO) (2014). Childhood Stunting: Challenges and opportunities. http://apps.who.int/iris/bitstream/10665/107026/1/WHO_NMH_NHD_GRS_14.1_eng.pdf.

[7] National Development Planning Commission, Republic of Ghana The Cost of Hunger in Africa, Ghana. Social and Economic Impact of Child Undernutrition on Ghana’s Long-Term Development. https://static1.squarespace.com/static/527789a2e4b0a23a823e44cd/t/57bc383eebbd1a30a1b5f412/1471953104396/GHANA_Report+FINAL.pdf.

[8] Dewey K.G., Begum K. (2011). Long-term consequences of stunting in early life: Long-term consequences of stunting. Matern. Child. Nutr..

[9] Ofori-Asenso R., Agyeman A.A., Laar A., Boateng D. (2016). Overweight and obesity epidemic in Ghana—A systematic review and meta-analysis. BMC Public Health.

[10] Centers for Disease Control and Prevention The Health Effects of Overweight and Obesity. https://www.cdc.gov/healthyweight/effects/index.html.

[11] Jones N.R.V., Conklin A.I., Suhrcke M., Monsivais P. (2014). The growing price gap between more and less healthy foods: Analysis of a novel longitudinal UK dataset. PLoS ONE.

[12] Darmon N., Ferguson E.L., Briend A. (2002). A Cost constraint alone has adverse effects on food selection and nutrient density: An analysis of human diets by linear programming. J. Nutr..

[13] World Bank Macro Poverty Outlook Ghana. http://pubdocs.worldbank.org/en/517001477329249847/pdf/mpo-am16-gha.pdf.

[14] Smith V.E. (1959). Linear Programming Models for the Determination of Palatable Human Diets. Am. J. Agric. Econ..

[15] Darmon N., Ferguson E., Briend A. (2002). Linear and nonlinear programming to optimize the nutrient density of a population’s diet: An example based on diets of preschool children in rural Malawi. Am. J. Clin. Nutr..

[16] Rambeloson Z.J., Darmon N., Ferguson E.L. (2008). Linear programming can help identify practical solutions to improve the nutritional quality of food aid. Public Health Nutr..

[17] Parlesak A., Geelhoed D., Robertson A. (2014). Toward the prevention of childhood undernutrition: Diet diversity strategies using locally produced food can overcome gaps in nutrient supply. Food Nutr. Bull..

[18] Food and Agriculture Organization of the United Nations Food-Based Dietary Guidelines. http://www.fao.org/nutrition/education/food-dietary-guidelines/home/en/.

[19] Ministry of Health, Ghana Dietary and Physical Activity Guidelines for Ghana, December 2009. http://alwag.org/education/courses/pa-guide.pdf.

[20] Parlesak A., Tetens I., Dejgård Jensen J., Smed S., Gabrijelčič Blenkuš M., Rayner M., Darmon N., Robertson A. Use of Linear Programming to Develop Cost-Minimized Nutritionally Adequate Health Promoting Food Baskets. http://journals.plos.org/plosone/article?id=10.1371/journal.pone.0163411.

[21] Food and Agriculture Organization of the United Nations West African Food Composition Table, 2012. http://www.fao.org/docrep/015/i2698b/i2698b00.pdf.

[22] Korkalo L., Hauta-Alus H., Mutanen M. (2011). Food composition Tables for Mozambique. http://www.helsinki.fi/food-and-environment/research/groups/Food_composition_tables_for_Mozambique.pdf.

[23] United States Department of Agriculture (USDA) USDA National Nutrient Database for Standard Reference, Release 28. https://www.ars.usda.gov/northeast-area/beltsville-md/beltsville-human-nutrition-research-center/nutrient-data-laboratory/docs/sr28-download-files/.

[24] Public Health England McCance and Widdowson’s The Composition of Foods Integrated Dataset 2015. https://www.gov.uk/government/publications/composition-of-foods-integrated-dataset-cofid.

[25] DTU Food, National Food Institute Denmark Danish Food Composition Databank. http://www.foodcomp.dk/v7/fcdb_download.asp.

[26] National Institute for Health and Welfare, Finland National Food Composition Database in Finland (Fineli). https://fineli.fi/fineli/en/index?.

[27] The Norwegian Food Safety Authority, University of Oslo Matvaretabellen. http://www.matvaretabellen.no/.

[28] Agunbiade S.O., Ojezele M.O., Alao O.O. (2015). Evaluation of the nutritional, phytochemical compositions and likely medicinal benefits of Vernomia amygdalina, Talinum triangulare and Ocimum basilicum leafy-vegetables. Adv. Biol. Res..

[29] Annan-Prah A., Agyeman J.A. (1997). Nutrient content and survival of selected pathogenic bacteria in kenkey used as a weaning food in Ghana. Acta Trop..

[30] Food and Agricultural Organization of the United Nations, United Nations University, World Health Organization (2001). Food and Nutrition Technical Report Series: Human Energy Requirements. http://www.fao.org/3/a-y5686e.pdf.

[31] World Health Organization, Food and Agriculture Organization of the United Nations (2004). Vitamin and Mineral Requirements in Human Nutrition. http://apps.who.int/iris/bitstream/10665/42716/1/9241546123.pdf?ua=1.

[32] Ghana Statistical Service, 2011. https://dhsprogram.com/pubs/pdf/FR262/FR262.pdf.

[33] Dantzig G.B., Koopmans T.C. (1951). Maximization of a linear function of variables subject to linear inequalities. Activity Analysis of Production and Allocation.

[34] Mason A.J., Klatte D., Lüthi H.-J., Schmedders K. (2012). OpenSolver—An open source add-in to solve linear and integer programmes in Excel. Operations Research Proceedings 2011.

[35] Food and Agricultural Organization of the United Nations Food Balance Sheets, FAOSTAT Dataset. http://www.fao.org/faostat/en/?#data/FBS.

[36] Ghana Statistical Service Ghana Living Standards Survey 6 (With a Labour Force Module) 2012-2013, Round Six (GLSS6). http://www.statsghana.gov.gh/docfiles/glss6/GLSS6_Main%20Report.pdf.

[37] Van den Broek N., Dou L., Othman M., Neilson J.P., Gates S., Gülmezoglu A.M., The Cochrane Collaboration (2010). Vitamin A supplementation during pregnancy for maternal and newborn outcomes. Cochrane Database of Systematic Reviews.

[38] Alhassan A., Adam A., Nangkuu D. (2017). Prevalence of neural tube defect and hydrocephalus in Northern Ghana. J. Med. Biomed. Sci..

[39] Bosu W.K. (2010). Epidemic of hypertension in Ghana: A systematic review. BMC Public Health.

[40] Roth G.A., Forouzanfar M.H., Moran A.E., Barber R., Nguyen G., Feigin V.L., Naghavi M., Mensah G.A., Murray C.J.L. (2015). Demographic and Epidemiologic Drivers of Global Cardiovascular Mortality. N. Engl. J. Med..

[41] Darko A.F., Allen B., Mazunda J., Rahimzai R., Dobbins C. (2013). Cost-minimizing food budgets in Ghana. J. Dev. Agric. Econ..

[42] Darmon N., Drewnowski A. (2015). Contribution of food prices and diet cost to socioeconomic disparities in diet quality and health: A systematic review and analysis. Nutr. Rev..

[43] Matallana González M.C., Martínez-Tomé M.J., Torija Isasa M.E. (2010). Nitrate and nitrite content in organically cultivated vegetables. Food Addit. Contam. Part B.

[44] Laar A.K., Aryeetey R.N.O., Mpereh M., Zotor F.B. (2017). Improving nutrition-sensitivity of social protection programmes in Ghana. Proc. Nutr. Soc..

